# Diagnostic Accuracy of Different Criteria of Pharmaco‐penile Duplex Sonography for Venous Erectile Dysfunction

**DOI:** 10.1002/jum.14982

**Published:** 2019-03-06

**Authors:** Li Chen, Lingling Xu, Jin Wang, Hong Li, Danqing Zhang, Cuihong Zhang, Huijun Jia, Mingxing Xie, Zhaohui Zhu, Yali Yang

**Affiliations:** ^1^ Department of Ultrasound Union Hospital, Tongji Medical College, Huazhong University of Science and Technology Wuhan China; ^2^ Department of Ultrasound The First People's Hospital of Yibin Sichuan China; ^3^ Hubei Province Key Laboratory of Molecular Imaging Wuhan China; ^4^ Department of Urology Surgery, West Campus, Union Hospital Tongji Medical College, Huazhong University of Science and Technology Wuhan China

**Keywords:** cavernosography, Doppler ultrasound, erectile dysfunction, intracavernous injection

## Abstract

**Objectives:**

The aim of this study was to analyze the diagnostic accuracy of different criteria of pharmaco‐penile duplex sonography in venous erectile dysfunction (ED).

**Methods:**

The following parameters were measured after an intracavernous injection test in patients with ED from May 2016 to February 2017 at our hospital: diameter, peak systolic velocity, end‐diastolic velocity, and resistance index of the cavernous artery; diameter and peak velocity (if leak occurred) of the deep dorsal vein. Three ultrasonographic diagnostic criteria of venous ED were applied. Criterion A: continuous blood flow signals in the deep dorsal vein, peak velocity greater than 3 cm/s, peak systolic velocity greater than 30 cm/s, end‐diastolic velocity greater than 5 cm/s; Criterion B: resistance index less than 0.89 and other parameters corresponding with Criterion A; Criterion C: resistance index less than 0.80 and other parameters corresponding with Criterion A. The diagnostic results of each criterion were compared with the cavernosographic results.

**Results:**

Thirty‐six patients were diagnosed as venous ED by cavernosography in 54 ED cases. The diagnostic specificity, sensitivity, and accuracy of Criterion A were 70.6%, 91.7%, and 84.9%, respectively. Those of Criterion B were 82.4%, 69.4%, and 73.6%, while the results for Criterion C were 94.1%, 33.3%, and 52.8%, respectively. Criterion A had the highest diagnostic accuracy, the largest area under the receiver operating characteristic curve (area = 0.811), and the highest consistency (kappa = 0.642) with the cavernosographic results in the 3 criteria. The difference was statistically significant (*P* < .05).

**Conclusions:**

Among the 3 commonly used ultrasonographic criteria, Criterion A is most appropriate in the diagnosis of venous ED.

**Abbreviations**
AUC: area under the receiver operating characteristic curve
CG: cavernosography
ED: erectile dysfunction
EDV: end‐diastolic velocity
ICI: intracavernous injection
PPDS: pharmaco‐penile duplex sonography
PSV: peak systolic velocity
RI: resistance index

Inadequate penile erection, also known as erectile dysfunction (ED), seriously affects the life quality of patients and is not conducive to marital affection and social harmony. According to the World Health Organization, there were about 150 million patients with ED worldwide in 2002, with an estimated 322 million by 2025.^1^ Venous type due to penile venous reflux accounts for more than 60% of ED^2^ and can be improved or even cured by timely intervention. Therefore, accurate assessment of penile hemodynamics is significantly important for clinical diagnosis and decision making in patients with venous ED.

Pharmaco‐penile duplex sonography (PPDS) observes penile hemodynamics before and after penile erection induced by intracavernous injection of vasoactive drugs (ICI test). It is a first‐line diagnostic modality of venous ED and widely used in clinical settings because it is safe and radiation free and allows for the observation of hemodynamic changes continuously and repeatedly compared with the traditional “gold standard” cavernosography (CG).^3^ There are, however, several different ultrasonographic diagnostic criteria for venous ED, but no consensus has presently been reached in academia worldwide.^4^ This study aimed to evaluate the accuracy of several of the most commonly used ultrasonographic criteria in the diagnosis of venous ED and to compare their results with those of CG to determine the best diagnostic criterion.

## Materials and Methods

### *Patient Population*

This was a prospective study conducted at Wuhan Union Hospital. Ethical approval was given by the Medical Ethics Committee of Tongji Medical College, Huazhong University of Science and Technology (IORG No. IORG0003571). Informed consent was obtained from patients before they were enrolled in the study.

From May 2016 to February 2017, patients with ED assessed by the International Index of Erectile Function–5 were selected at our hospital. Inclusion criteria were as follows: no suggestion of penile malformation, endocrine system disease, or high hemodynamic status by history, physical examination, routine blood tests, or hormonal tests; no psychogenic factors as assessed by the Symptom Checklist–90 scores; and maximum penile erection hardness of Grade III or higher after the ICI test. We used the Erection Hardness Score to evaluate the erectile hardness of the penis. Grade I indicates that the penis is larger but not hard; Grade II, hard but not hard enough for penetration; Grade III, hard enough for penetration but not completely hard; Grade IV, completely hard and fully rigid.^5^ Patients with hypertension were not included, and there was also no age restriction in our study. The patients were recruited consecutively. All patients were subject to detailed PPDS and CG examinations.

### *Pharmaco‐penile Duplex Sonography*

All patients underwent a complete ultrasonographic examination using an IE 33 ultrasound system (Philips Healthcare, Andover, MA) with a 5‐ to 12‐MHz probe.

**Figure 1:**
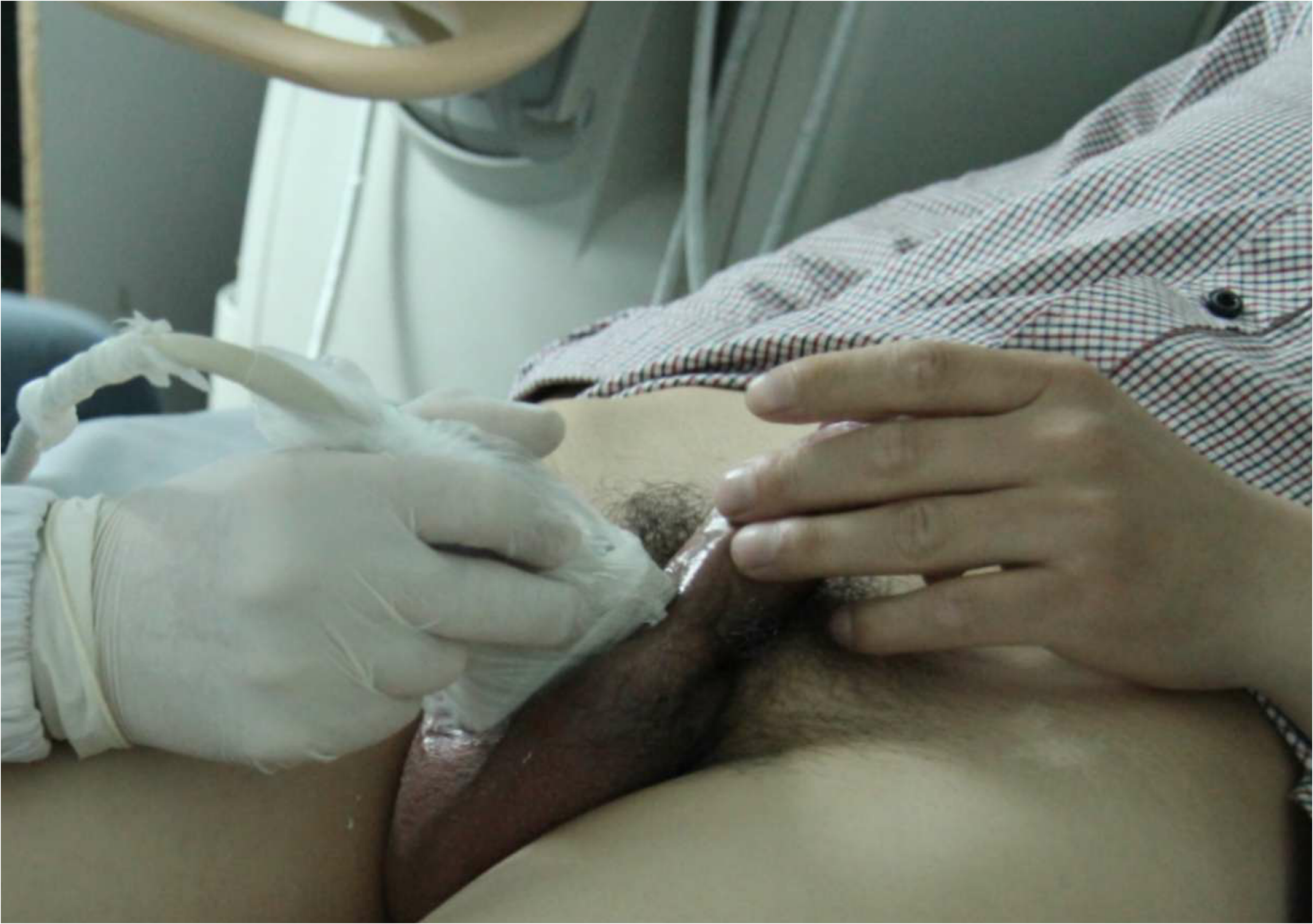
Actual operation photo of pharmaco‐penile duplex sonography.

Patients were asked to lie in a supine position, and their perineal areas were sterilized and covered with sterile sheet. The patient was instructed to hold the penis with his right hand and gently rest it against the abdominal wall. The probe was placed at the base of the penis, which was scanned from the root to the glans with continuous transverse and longitudinal sections to observe the penile anatomy (Figure 1). The ICI test was then given. After routine disinfecting and towel spreading, the base of the penis was tied with a rubber tourniquet. Twenty micrograms of prostaglandin E1 was injected into one side of the cavernosum, and the penis was then kneaded gently to help the drug spread quickly. The tourniquet was removed, and then the maximum hardness of the penis was evaluated. Sexual fantasies and local stimuli could be ordered if necessary to achieve maximal rigidity.

**Figure 2:**
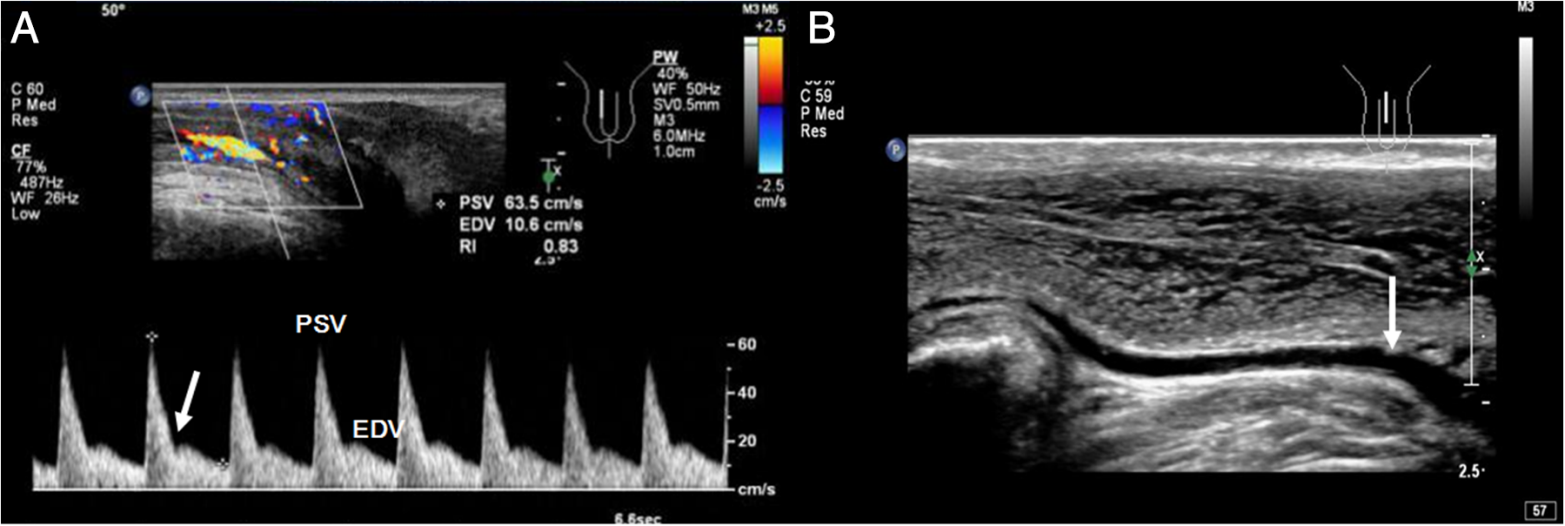
Normal ultrasound images in a 24‐year‐old man. **A,** Blood flow spectrum of the cavernous artery at the end of phase II with a characteristic of a waveform notch (arrow) at end systole. **B,** Measurement site (arrow) of the deep dorsal vein at the base of the penis.

About 5 to 10 minutes after injection, the sampling gate (size, 0.5 mm) was placed at approximately l to 2 cm from the origins of the bilateral cavernous arteries and their diameters measured, and the average was recorded.^6^ The spectrums of cavernosal arteries were observed repeatedly at the same sampling locations as mentioned above, respectively, taking care to keep the angle between the sampling line and the longitudinal axis of the blood vessel less than 60 degrees.^7^ Peak systolic velocity (PSV), end‐diastolic velocity (EDV), and resistance index (RI) were measured in the end‐phase II arterial spectrum (Figure 2A). The deep dorsal vein diameter and peak velocity (if leak occurred) were measured at the base of the penis (Figure 2B). At least 5 cycles were included in each spectrum image, and all parameter measurements were repeated 3 times to average. After examination, the patient was observed and discharged from the department only after the erection subsided with no significant discomfort.

The following 3 ultrasonographic diagnostic criteria were used, respectively, to diagnose venous ED:

- Criterion A: Continuous blood flow signals in the deep dorsal vein, peak velocity greater than 3 cm/s, PSV greater than 30 cm/s, and EDV greater than 5 cm/s^8^, ^9^
- Criterion B: RI less than 0.89 and other parameters corresponding with Criterion A^10^, ^11^
- Criterion C: RI less than 0.80 and other parameters corresponding with Criterion A^12^, ^13^, ^14^

### *Cavernosography*

All patients were also undergoing a CG examination using a digital subtraction angiography instrument (AXIOM Artic DFC; Siemens, Munich, Germany) and a double‐cylinder syringe (Dongjia 2000, Jiangsu, China). Supine, lateral, and oblique positions were all required for the ICI test. After the penis was fully erect, all patients were intracavernously injected with 30 to 100 mL of iodixanol injection (Visipaque 320; GE Healthcare, Chalfont St. Giles, England) with 10 to 90 mL/min of injection velocity. Posteroanterior images were taken as well as 45‐degree oblique radiographs, and then the position of the venous leak was observed. Venous ED was diagnosed while venous drainage of the penis was significantly enhanced by contrast agent. Absent or faint enhancement of venous drainage was considered as negative for venous ED.

The Doppler ultrasonography was performed by an ultrasound physician, and the cavernography was performed by a radiologist. To eliminate bias, they did not know each other's test results.

### *Repeatability Test*

Twenty subjects were randomly selected from the group after 2 weeks. To assess internal consistency, PSVs were measured from this group and compared with the previous results by the same observer, who had more than 4 years’ experience in ultrasound diagnosis. To assess the interobserver consistency, another observer with the same or greater diagnostic experience then measured the PSVs in a single‐blind condition.

### *Statistical Analysis*

Statistical analyses were performed with SPSS version 18.0 software (IBM Corporation, Armonk, NY). Descriptive analysis of all data with a normal distribution was expressed as mean ± standard deviation, and the count data were expressed as frequency and composition ratio. A *t* test was conducted to compare the parameters of the venous ED group and nonvenous ED group. Sensitivity, specificity, accuracy, positive predictive value, and negative predictive value were performed to assess the accuracy of different diagnostic criteria. Consistency in the diagnostic results between PPDS and CG was analyzed with the kappa test. The area under the receiver operating characteristic curve (AUC) was used to compare the accuracy of different criteria. Statistical significance was accepted when the *P* value was less than 0.05. The intraobserver and interobserver consistency were analyzed with a Bland‐Altman method.

## Results

Fifty‐three patients with ED were enrolled, and all underwent complete PPDS and CG examination successfully. Patients’ ages ranged from 17 to 59 years, with a mean of 29.79 ± 6.91 years. No abnormal erections occurred during PPDS examination. Among them, 21 subjects complained of local distention and mild pain after injection, which alleviated spontaneously within 2 hours. Local congestion and edema occurred in 1 case but was relieved after 1 week of hospitalization.

**Figure 3:**
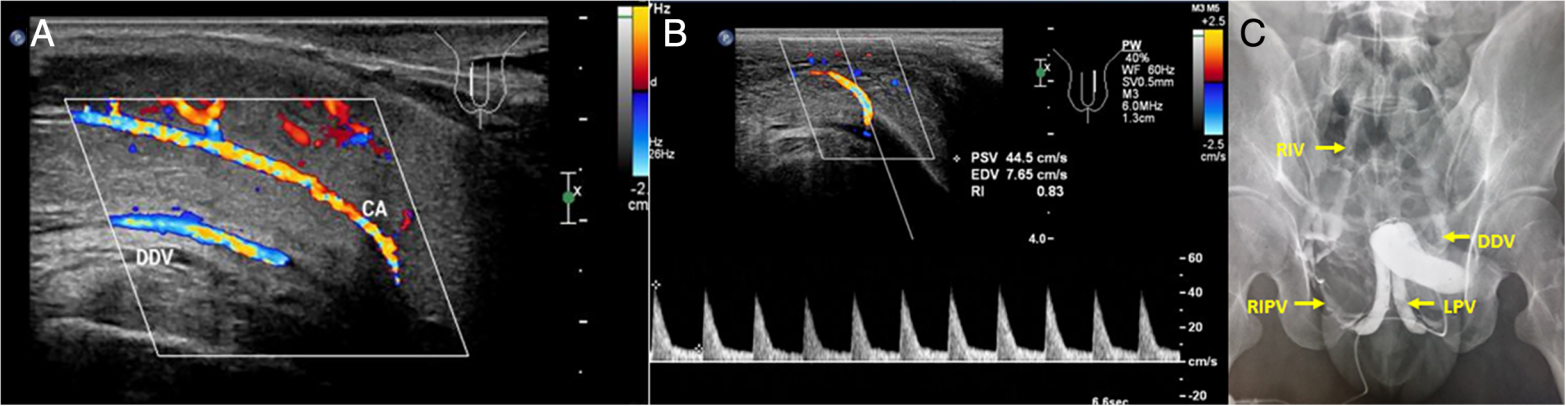
Mixed venous leak in a 26‐year‐old man. **A,** The continuous blood flow signal was observed in the deep dorsal vein (DDV) after intracavernosal injection by ultrasonography. **B,** The cavernous arterial (CA) spectrum showed PSV > 30 cm/s, EDV > 5 cm/s, and RI = 0.83. **C,** The contrast agent can be seen in the deep dorsal vein, the bilateral peduncular veins, the bilateral internal pudendal veins, the bilateral iliac veins, and even the right external iliac vein in the cavernographic image. DDV indicates deep dorsal vein; EDV, end‐diastolic velocity; LPV, left peduncular vein; PSV, peak systolic velocity; RIPV, right internal pudendal vein; RIV, right iliac vein.

**Figure 4:**
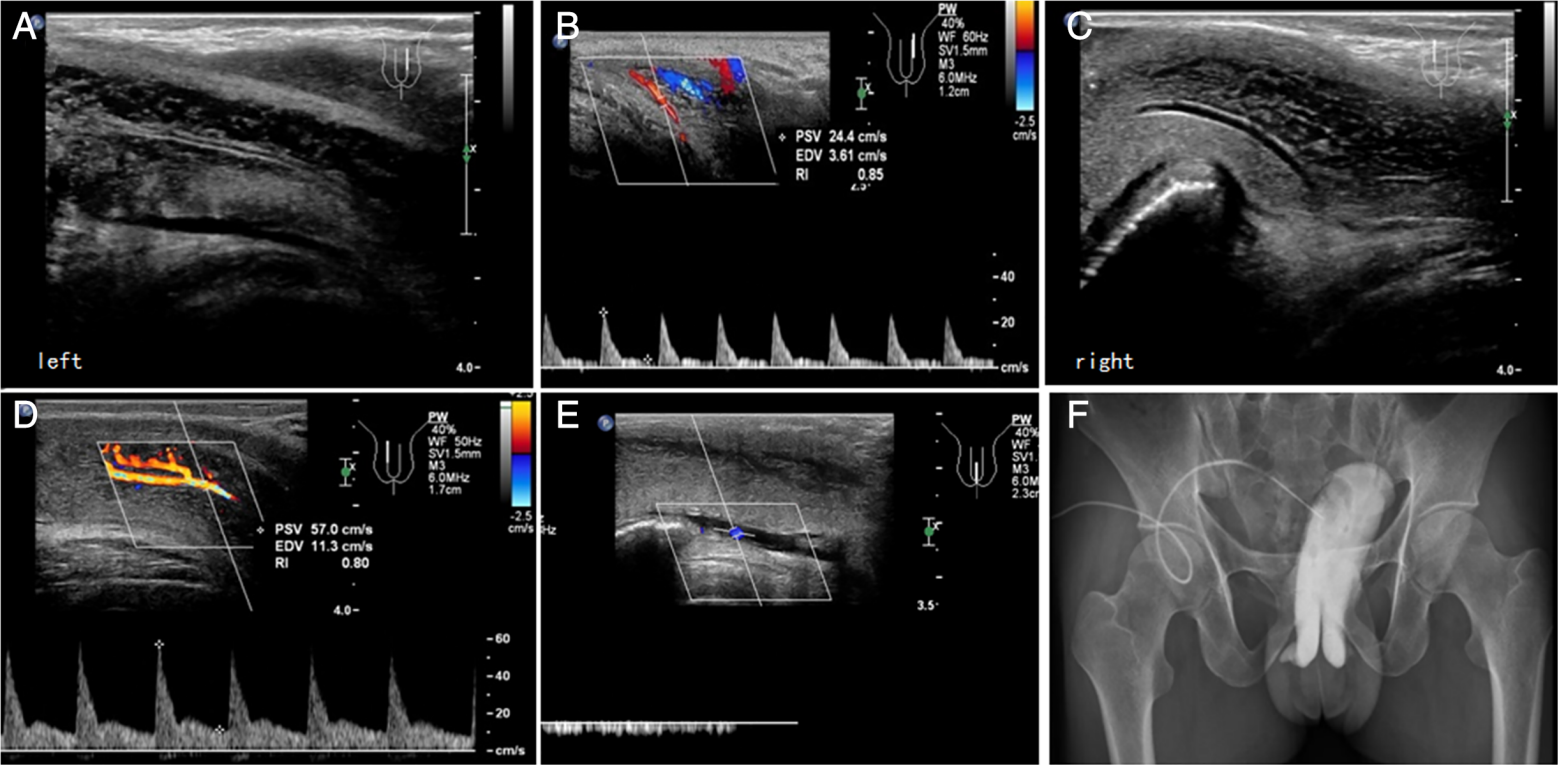
No cavernosal leakage in another 26‐year‐old man who had a false‐positive diagnosis of PPDS. **A and B,** The spectrum of the left cavernous artery that had a finer lumen suggested PSV < 30 cm/s, EDV < 5 cm/s, and RI = 0.85. **C and D,** The spectrum of the right cavernous artery that had a wider lumen suggested PSV > 30 cm/s, EDV > 5 cm/s, and RI = 0.80. **E,** The spectrum of the deep dorsal vein revealed a low‐velocity discontinuous blood flow. **F,** The cavernosography showed no obvious enhancement of the penile venous drainage. EDV indicates end‐diastolic velocity; PPDS, pharmaco‐penile duplex sonography; PSV, peak systolic velocity; RI, resistance index.

Thirty‐six patients were diagnosed as venous ED by CG examination, accounting for 67.9% of 53 cases, including 33 mixed venous leaks (of which deep dorsal venous leak was present in 32 patients, accounting for 88.9% of all venous ED) and 3 peduncular venous leaks (Figure 3). The other 17 patients were finally excluded as venous ED by CG, representing minor dorsal deep venous leak (n = 8) or no venous leak (n = 9) (Figure 4).

### *Comparison of Ultrasonic Parameters Between Venous ED and Nonvenous ED*

The patients were divided into the venous ED group (n = 36) and nonvenous ED group (n = 17) according to the results of CG examination. The ultrasonographic parameters of the 2 groups are shown in Table 1. Compared with the nonvenous ED patients, patients with venous ED had increased diameter (*P* < 0.05) and evidently increased peak velocity of blood flow (*P* < 0.001) of the deep dorsal vein. However, there was no significant difference in the diameter, PSV, EDV, or RI of the cavernous artery (all *P* > 0.05).

**Table 1:** Ultrasonographic Parameters of the Venous ED Group and Nonvenous ED Group

Ultrasonographic Parameters	Venous ED (n = 36)	Nonvenous ED (n = 17)	*P*
D (mm)	1.25 ± 0.09	1.29 ± 0.7	.053
PSV (cm/s)	48.36 ± 9.88	51.59 ± 10.97	.295
EDV (cm/s)	6.72 ± 3.06	6.74 ± 2.66	.978
RI	0.82 ± 0.06	0.83 ± 0.07	.701
D′ (mm)	3.25 ± 0.23	3.41 ± 0.27	.032
V (cm/s)	5.89 ± 2.96	1.68 ± 2.81	<.001

D, diameter of the cavernous artery; D′, diameter of the deep dorsal vein; ED, erectile dysfunction; EDV, end‐diastolic velocity; PSV, peak systolic velocity; RI, resistance index; V, peak velocity of the deep dorsal vein.

### *Diagnostic Accuracy of Different PPDS Criteria*

Comparison of statistical accuracy indicators between the diagnostic results of the 3 ultrasound criteria and the CG are summarized in Table 2.

**Table 2:** Statistical Results of Diagnostic Accuracy of Different Ultrasound Criteria

Statistical Indices	Criterion A	Criterion B	Criterion C
Specificity (%)	70.59	82.35	94.12
Sensibility (%)	91.67	69.44	33.33
Accuracy (%)	84.91	73.58	52.83
Positive predictive value (%)	86.84	89.29	92.31
Negative predictive value (%)	80.00	56.00	40.00

The consistency of diagnostic results between PPDS with different criteria and CG is shown in Table 3. The results showed a moderate consistency between the qualitative diagnostic results of ultrasound Criteria A and B versus the CG results (0.4 ≤ k < 0.75), and the consistency of Criterion A was significantly higher than that of Criterion B (E < 0.05), whereas the consistency between the results of ultrasound Criterion C and CG was poor (k < 0.4).

**Table 3:** Kappa Test of Diagnostic Results Between PPDS With Different Criteria and CG

	Approx. Sig	Kappa Value	Exact Sig. (2‐Sided)
Criterion A	0.000	0.642	0.727
Criterion B	0.000	0.461	0.057
Criterion C	0.030	0.202	0.000

CG, cavernosography; PPDS, pharmaco‐penile duplex sonography.

**Figure 5:**
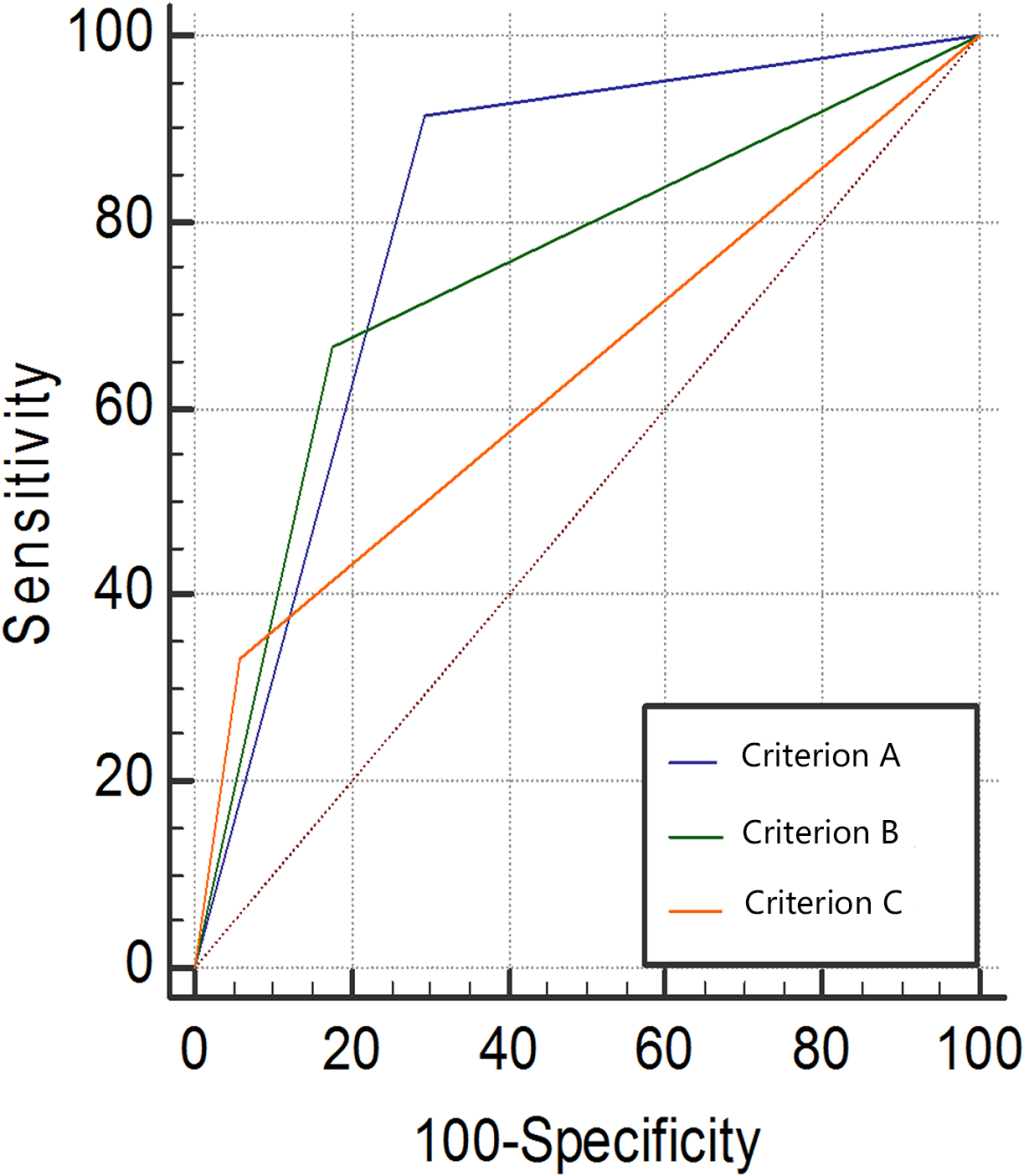
Receiver operating characteristic curves of different diagnostic criteria of pharmaco‐penile duplex sonography for venous erectile dysfunction.

The receiver operating characteristic curve of each criterion (Figure 5) and the AUC results listed in Table 4 suggested that the AUC of Criterion A was the largest, thus indicating the highest diagnostic accuracy.

**Table 4:** Area Under the ROC Curve of Different Ultrasound Diagnostic Criteria

				Progressive 95% Confidence Interval	
Variable	Area	Standard Error^a^	Progressive Sig.^b^	Lower Limit	Upper Limit
Criterion A	0.811	0.072	0.000	0.670	0.952
Criterion B	0.745	0.072	0.004	0.604	0.886
Criterion C	0.637	0.077	0.109	0.109	0.788

a Nonparametric hypothesis;

b Null hypothesis: area under the solid line = 0.5.

In addition, 2 patients were diagnosed by ultrasonography with penile sclerosis besides the vascular lesions. One had a history of penile trauma and a deformity of penile curvature. The ultrasonographic images showed obviously uneven texture and circular calcification of his cavernous body and thickening, enhancing, and stiffness of his tunica albuginea, while continuous blood flow signal was also observed in the deep dorsal vein after drug injection.

### *Consistency of Ultrasonographic Measurement*

**Figure 6:**
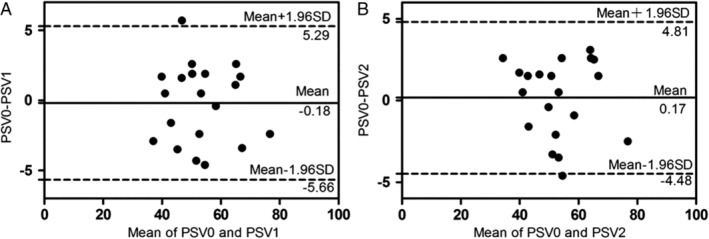
The Bland‐Altman plots for peak systolic velocity measurement:**A,** intraobserver consistency; **B,** interobserver consistency.

The Bland‐Altman analysis of PSV measurement (Figure 6) indicated that the intraobserver and interobserver coefficients of variation were 19.6% and 19.7%, respectively. The 95% confidence intervals between and within observers were –5.66 to 5.29 and –4.48 to 4.81, respectively, with 95% (19of 20) of the points ranging in the 95% consistency limit (Figure 5). These results revealed that the parameter measurement by ultrasonography had good repeatability and consistency between and within observers.

## Discussion

Penile erection is a complex physiologic process caused by the coordination of psychosocial, hormonal, neurologic, and hemodynamic factors.^15^ Hemodynamic changes during the erection include an increase in penile arterial blood flow, restriction of cavernous venous drainage, and increase in intracavernosal pressure. Any disruption to the dynamic balance of blood flows in the cavernous body would lead to failure of penile erection. Among them, penile venous leak plays a major role in the cause of ED. The most important mechanisms include smooth muscle defect of the cavernous sinus leading to dysfunction of sinusoidal expansion and venous occlusion, dysplasia of subalbuminous venous plexus, or abnormal drainage between the corpus cavernosum and the urethral cavernous body.^16^ It was reported that venous ED accounts for more than 60% of ED patients,^2^ and the proportion in our study was 67.9%, which is thus consistent with previous studies.

Cavernosography is the gold standard for diagnosing venous ED. It can provide reliable evidence for surgical treatment by identifying the location and the pattern of venous reflux. However, this method is invasive, time consuming, expensive, and will cause some pain, which is not conducive to follow‐up of disease conditions and monitoring of therapeutic effects. Pharmaco‐penile duplex sonography has been developed in recent years as a good method to evaluate the function of penile vessels. Based on the traditional evaluation of penile anatomy, PPDS induces penile erection by injecting vasoactive drugs into the cavernous body (ICI test) and quantitatively evaluates by color Doppler ultrasonography the blood flow information of the penile arteries and veins before and after erection. It can directly observe whether a reflux in the deep dorsal vein occurs after erection in real time. The parameters of cavernosal arteries, including PSV, EDV, and RI, reflect the situation of venous reflux. Thus, PPDS could also assess penile venous leakage indirectly by observing these arteries. Coupled with the technical advantages of ultrasonography, PPDS's safety, radiation‐free nature, repeatability, and ability to continuously and dynamically observe hemodynamic parameters have made it one of the most valuable methods for diagnosing vascular ED.^17^ The disadvantage of PPDS is that it is not able to directly capture panoramic and stereoscopic images and requires high proficiency of the operators.^18^, ^19^

**Figure 7:**
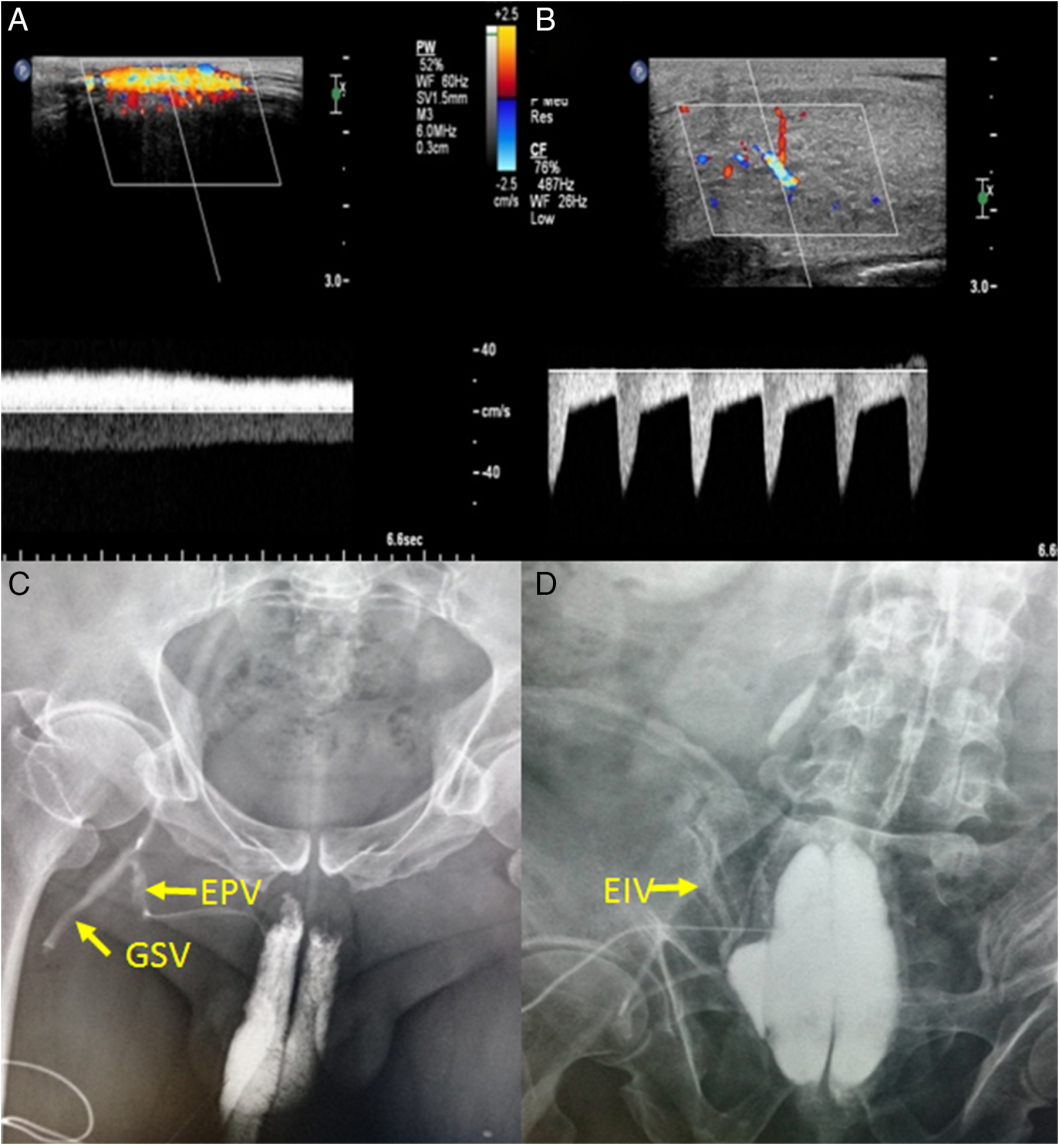
Cavernosal leakage of the penis in a 54‐year‐old‐man, mostly occurring in the external pudendal vein, who underwent a segmented ligation of the deep dorsal vein 2 years ago. **A and B,** The ultrasonographic images showed continuous blood flow signal in the superficial dorsal vein but no signal in the deep dorsal vein. The spectrum of the cavernous artery showed normal PSV, EDV > 7 cm/s, and RI < 0.80. He was finally diagnosed with superficial dorsal venous leak by ultrasound. **C and D,** The cavernosography showed contrast enhancement of the external pudendal vein, the great saphenous vein, and the external iliac vein on the right side. EDV indicates end‐diastolic velocity; EIV, external iliac vein; EPV, external pudendal vein; GSV, great saphenous vein; PSV, peak systolic velocity; RI, resistance index.

Continuous blood flow within the venous drainage pathway in the erect state is direct evidence for the diagnosis of penile venous leak. There are superficial, middle, and deep layers of the drainage system in the penile vein.^20^ The superficial veins course between the Colles fascia and the Buck fascia, are incorporated into a superficial dorsal vein at the back of the penis, and finally drain into the great saphenous vein. The drainage pathway of the middle layer of the penile veins is from the emissary vein to the circumflex vein, then to the deep dorsal penile vein, and finally merging into the prostatic venous plexus. The deep layer of the veins includes the peduncular vein, the cavernous vein, and the internal pudendal vein from far to near. In the above penile venous drainage system, the deep dorsal penile vein (middle layer) plays a dominant role. In our study, 89% (32 of 36) of venous ED involved the dorsal vein, while other reports reveal proportions of approximately 75% to 78%.^21^ Ultrasonography mainly evaluates the drainage of the deep dorsal vein. In addition, the leak of the superficial dorsal vein can also be assessed, which presents continuous blood flow in the superficial dorsal vein after erection (Figure 7). However, it is difficult to display the deep layer of penile veins, such as the peduncular vein, which is an important reason for missed diagnosis using PPDS.

### *Diagnostic Value of RI*

At present, the commonly used indicators of PPDS in the diagnosis of venous ED include the PSV, EDV, and RI parameters of the penile cavernous artery and whether there is continuous blood flow in the deep dorsal vein and, if it exists, its peak value. However, the methods and diagnostic criteria of ultrasound examination are variable in the clinic without an acknowledged diagnostic guideline or recommendation. Most European reports adopt either Criterion A or Criterion B, that is, continuous blood flow signals in the deep dorsal vein, velocity greater than 3 cm/s, PSV greater than 30 cm/s, and EDV greater than 5 cm/s of the cavernosal artery, with RI less than 0.89 or without the limit of RI value. In China, Criterion C is usually used, which is consistent with Criterion A or B, except for the standard of RI less than 0.80. Some studies^20^, ^21^ also suggested RI less than 1 as one of the standards. However, RI in these studies was usually measured 5 to 10 minutes after the drug injection. During this time, the hemodynamics of the cavernous artery presented the characteristics of the end of phase II, in which RI greater than 1 is noted in almost all of the ED patients. Thus, this kind of criterion is not listed separately in our study due to similarity to Criterion A.

After penile ICI test, the pressure in the cavernous body cannot be maintained if there is a venous leak, although the perfusion of the penile artery is not reduced significantly. The blood flow of the cavernous artery in patients with venous ED is in a high‐speed and low‐resistance condition, characterized as PSV greater than 30 cm/s and EDV greater than 5 cm/s on the arterial spectrum. The RI indirectly reflects the blood flow of the cavernous artery and distal microcirculation (composed of spiral arterioles, cavernous spaces, and venules), which theoretically should be decreased. This is why some researchers use RI less than 0.80 or RI less than 0.89 as one sign of venous leak. However, this is not the case in our study. In some patients with venous ED diagnosed by CG, RI was still high (>0.80 or >0.89) at the end of phase II. It seems that the RI limitation of Criterion B or C led to misdiagnosis by ultrasonography instead. In contrast, Criterion A without RI limitation has the largest AUC, the highest sensitivity (91.7%), and the highest accuracy (84.9%), which is most valuable among the 3 diagnostic criteria. Yang et al^21^ reported similar results. In their study, the sensitivity, specificity, and accuracy of PPDS were 100%, 77.2%, and 77.2%, respectively, using Criterion A to diagnose venous ED compared with CG.

Based on the literature reports and the results of our study, we consider that the reason the RI value of some patients with penile venous leak does not decrease may be related to several factors. First, the diameters of the bilateral cavernous arteries are significantly different in some patients, with RI decreased in the wider artery, while it is increased in the narrower one. In this study, the RI values of both sides were measured and the average was taken, thus resulting in a higher RI value (Figure 4). Second, in some patients, the cavernous artery was tortuous, dysplastic, or complicated with atherosclerotic plaques, leading to cavernous arterial stenosis and then the higher PSV, thus obtaining higher RI values. Once again, the drug dosage, environment, and psychological state of patients affected erectile hardness, eventually affecting the results of ultrasound examination. We also consider that the operating inconsistency of ultrasound examiners in measuring the blood flow spectrum of the cavernous artery, including sampling site, sampling time, and angle of sound beam and bloodstream, affected the RI values to some extent.

### *Methods of Improving the Diagnostic Accuracy of Ultrasonography*

In the diagnosis of vascular ED by PPDS, the parameters are affected by penile hardness, sampling site, and sampling time. Based on our experiences, the following operating specifications could improve diagnostic accuracy:

- Keep the hardness of the penis in PPDS consistent with that in CG when obtaining the parameters after injection. In this study, the penis was required to achieve an erectile hardness up to Grade 4 with CG, whereas a Grade 3 or greater degree of hardness was acceptable when measuring with PPDS. This reduction in the requirement for erectile hardness may have an impact on the final diagnosis by ultrasonography.
- Ensure that the dorsal side of the penis is attached to the abdominal wall and place the transducer near the pubic symphysis when measuring the deep dorsal vein (Figure 1). The vein is lightly oppressed in this place with a narrower lumen, and blood flow is increased, thus resulting in easier observation and acquisition of the color signal of the associated venous leakage (Figure 2B).
- Measure the cavernous arteries at the beginning segments because of their various courses, and then attempt to minimize the measurement angle between sound beam and bloodstream.
- Venous ED cannot be completely excluded even if the deep dorsal vein shows no significant sustained blood flow signal. The parameters of the cavernous artery play an important role in diagnosis, which suggests measurement during the end of phase II of the arterial spectrum.

### *Effect of PPDS Accuracy on Clinical Management*

Pharmaco‐penile duplex sonography can not only visualize the deep dorsal vein leakage but also indirectly evaluate the penile artery insufficiency by observing the penile erectile hardness and ultrasonographic hemodynamics after the ICI procedure. It can make up for the insufficiency of CG, which cannot show the arterial blood supply. In patients with venous leakage combined with arterial insufficiency, the penis is usually not able to achieve Grade III to IV erection. The sum of PSVs of the left and right cavernous arteries is also less than 50 cm/s. On the contrary, Grade III to IV erection can be easily achieved in patients without penile artery insufficiency, and the sum of PSVs of bilateral artery is more than 50 cm/s.^22^

It is very important for clinical decision making whether patients with venous leakage also have arterial insufficiency. For patients with simple venous leakage, surgical ligation or medication to increase the arterial blood supply can be used. If the patient has insufficient arterial blood supply, drugs and vacuum negative pressure should be used to increase the arterial blood supply, and the venous leakage should be treated surgically.

Pharmaco‐penile duplex sonography is widely used in clinics. After eliminating penile malformations, endocrine system diseases, and hyperhemodynamic states, PPDS can be performed to exclude different types of vascular ED, thus providing the best clinical treatment.

There were limitations to this study. First, the number of cases observed in this study was relatively small and failed to address the variability of results due to operator dependence. Second, although there was no age limit in this study, most of the patients were younger. Therefore, the results of this study in middle‐aged and elderly patients with ED needed to be further explored. Third, if the arterial blood supply was insufficient, most of them could not maintain Grade III hardness to perform the ICI test. Our research was mainly aimed at venous ED, while mixed and arteriogenic ED remain to be further studied.

In conclusion, PPDS has the ability to detect a continuous leak of the penile deep dorsal vein and can evaluate the parameters of the cavernous arteries and veins before and after the ICI test. Therefore, it is an applicable method for diagnosing venous ED. The comparison of various commonly used ultrasound criteria suggests that the diagnostic accuracy is higher without an RI limit. In addition, by providing information on penile anatomy, ultrasonography can also help to diagnose other penile diseases, such as dysplasia, trauma, tumor, and so on. With the introduction of recognized operation standards and diagnostic guidelines in the future, ultrasonography will play a greater role in etiologic diagnosis, intraoperative monitoring, and assessment of the curative effect in patients with ED.

## References

[1] Chew KK , Stuckey B , Bremner A , Earle C , Jamrozik K . Male erectile dysfunction: its prevalence in western Australia and associated sociodemographic factors. J Sex Med 2008; 5:60–69. 17645447 10.1111/j.1743-6109.2007.00548.x

[2] Patel DV , Halls J , Patel U . Investigation of erectile dysfunction. Br J Radiol 2012; 85(Spec No 1):S69–S78. 23118101 10.1259/bjr/20361140 PMC3746402

[3] Xuan XJ , Bai G , Zhang CX , et al. The application of color Doppler flow imaging in the diagnosis and therapeutic effect evaluation of erectile dysfunction. Asian J Androl 2016; 18:118–122. 25994651 10.4103/1008-682X.155533 PMC4736339

[4] Sikka SC , Hellstrom WJ , Brock G , Morales AM . Standardization of vascular assessment of erectile dysfunction: standard operating procedures for duplex ultrasound. J Sex Med 2013; 10:120–129. 10.1111/j.1743-6109.2012.02825.x 22970798

[5] Goldstein I , Lue TF , Padma‐Nathan H , et al.Oral sildenafil in the treatment of erectile dysfunction. Sidenafil Study Group. N Engl J Med 1998; 338:1397–1404. 9580646 10.1056/NEJM199805143382001

[6] Pagano MJ , Stahl PJ . Variation in penile hemodynamics by anatomic location of cavernosal artery imaging in penile duplex Doppler ultrasound. J Sex Med 2015; 12:1911–1919. 26177146 10.1111/jsm.12958

[7] Attia AA , Yasien HA , Abdullah MS , Abo Hola MS . How to avoid the false diagnosis of venous leakage by pharmaco‐penile duplex ultrasonography? Menoufia Med J 2017; 30:928–934.

[8] Papagiannopoulos D , Khare N , Nehra A . Evaluation of young men with organic erectile dysfunction. Asian J Androl 2015; 17:11–16. 25370205 10.4103/1008-682X.139253 PMC4291852

[9] Khanzada U , Khan SA , Hussain M , et al. Evaluation of the causes of erectile dysfunction in patients undergoing penile Doppler ultrasonography in Pakistan. World J Mens Health 2017; 35:22–27. 28459144 10.5534/wjmh.2017.35.1.22 PMC5419116

[10] Mutnuru PC , Ramanjaneyulu HK , Susarla R , Yarlagadda J , Devraj R , Palanisamy P . Pharmaco penile duplex ultrasonography in the evaluation of erectile dysfunction. J Clin Diagn Res 2017; 11:TC07. 10.7860/JCDR/2017/25092.9270 PMC5324466 28274021

[11] Quam JP , King BF , James EM , et al. Duplex and color Doppler sonographic evaluation of vasculogenic impotence. AJR Am J Roentgenol 1989; 153:1141–1147. 2683672 10.2214/ajr.153.6.1141

[12] Tang Y , Jiang X , Yang FJ , et al. Study on diagnostic value of Doppler ultrasonography in vascular erectile dysfunction. Chin J Androl 2006; 20:31–33.

[13] He Z , Chen M , Zhang K , Jin J . The value of dynamic color duplex scanning in the diagnosis of vascular erectile dysfunction. Nat J Androl 2006; 12:62–65. 16483164

[14] Li H , Liang P , Zhang J , Ou S , Xiao J . Application of colour Doppler ultrasonography after intracavernous injection in the diagnosis of erectile dysfunction. Chin J Mod Med 2007; 17:2400–2402.

[15] Shamloul R , Ghanem H . Erectile dysfunction. Lancet 2013; 381:153–165. 23040455 10.1016/S0140-6736(12)60520-0

[16] Virag R , Paul JF . New classification of anomalous venous drainage using caverno‐computed tomography in men with erectile dysfunction. J Sex Med 2011; 8:1439–1444. 21366881 10.1111/j.1743-6109.2011.02226.x

[17] Ismail A , Tabari AM , Alhasan SU , Abdullahi A. Dynamic and morphologic evaluation of erectile dysfunction on penile Doppler sonography and contrast cavernosography. Niger J Clin Pract 2017; 20:729–733. 28656928 10.4103/njcp.njcp_158_16

[18] Herwig R , Greilberger J , Weibl P . CT cavernosography and penile venous leak. JOJ Urol Nephrol 2017; 3:5555613.

[19] Aiyekomogbon JO , Igashi JB , Lawan RO , Bioku MJ , Ameadaji M . Colour Doppler sonography of the penis in the evaluation of erectile dysfunction: our experience in Abuja, Nigeria. Niger Postgrad Med J 2017; 24:210–216. 29355159 10.4103/npmj.npmj_144_17

[20] Hsu GL , Hung YP , Tsai MH , et al. The venous drainage of the corpora cavernosa in the human penis. Arab J Urol 2013; 11:384–391. 26558109 10.1016/j.aju.2013.04.002 PMC4443007

[21] Yang L , Wang Y , Song T , et al. The use of CDUS checking deep dorsal vein velocity in the diagnosis of erectile dysfunction. Chin J Ultrasound Med 2015; 31:57–59.

[22] Lopez JA , Espeland MA , Jarow JP . Interpretation and quantification of penile blood flow studies using duplex ultrasonography. J Urol 1991; 146:1271–1275. 1942277 10.1016/s0022-5347(17)38066-7

